# An efficient algorithm for estimating brain covariance networks

**DOI:** 10.1371/journal.pone.0198583

**Published:** 2018-07-12

**Authors:** Marcela I. Cespedes, James McGree, Christopher C. Drovandi, Kerrie Mengersen, James D. Doecke, Jurgen Fripp, for the Alzheimer’s Disease Neuroimaging Initiative

**Affiliations:** 1 School of Mathematical Sciences, Queensland University of Technology, Brisbane, Queensland, Australia; 2 Health and Biosecurity/ Australian e-Health Research Centre, CSIRO, Herston, Queensland, Australia; University of Texas at Austin, UNITED STATES

## Abstract

Often derived from partial correlations or many pairwise analyses, covariance networks represent the inter-relationships among regions and can reveal important topological structures in brain measures from healthy and pathological subjects. However both approaches are not consistent network estimators and are sensitive to the value of the tuning parameters. Here, we propose a consistent covariance network estimator by maximising the network likelihood (MNL) which is robust to the tuning parameter. We validate the consistency of our algorithm theoretically and via a simulation study, and contrast these results against two well-known approaches: the graphical LASSO (gLASSO) and Pearson pairwise correlations (PPC) over a range of tuning parameters. The MNL algorithm had a specificity equal to and greater than 0.94 for all sample sizes in the simulation study, and the sensitivity was shown to increase as the sample size increased. The gLASSO and PPC demonstrated a specificity-sensitivity trade-off over a range of values of tuning parameters highlighting the discrepancy in the results for misspecified values. Application of the MNL algorithm to the case study data showed a loss of connections between healthy and impaired groups, and improved ability to identify between lobe connectivity in contrast to gLASSO networks. In this work, we propose the MNL algorithm as an effective approach to find covariance brain networks, which can inform the organisational features in brain-wide analyses, particularly for large sample sizes.

## 1 Introduction

Brain networks derived from neuroimaging data have been shown to quantify the level of brain atrophy, and hence the relative stage of neurological disease and identify disease related changes [1]. Cortical networks from structural magnetic resonance imaging (MRI) consist of nodes which represent brain regions of interest (ROI) and edges that link two nodes if these regions have spatial correlation or similarity [2, 3]. Unlike networks from ROI volume or surface area, cortical thickness networks have been shown to be a more stable measure along the Alzheimer’s Disease (AD) continuum. This is because cortical thickness is a direct measure of cortical atrophy due to cytoarchitectural features of the cortex tissue [2]. The resultant networks characterise alterations in the communication processes across multiple ROI associated with morphological changes due to disease onset and progression [4–8]. Furthermore, network analysis of cortex connectivity maps allow for the detection of ROI that serve particular cognitive functions, thus providing a link between brain structure and function [9–11]. Such links include spatial topographical patterns typically observed between those with and without neurological disease [12–14].

An early approach to derive a cortical correlation network is the application of Pearson pairwise correlation (PPC) analyses for all possible pairs of ROIs [3, 15–19]. This approach quantifies the presence or absence of a linear relationship between two sets of observations, and a threshold (tuning parameters) is applied to the correlation values to produce the resultant network. In addition to being threshold dependent, another disadvantage of PPC networks is the reliance on correlations based on independent analysis among two ROIs. While these methods quantify the correlation between region pairs *i* and *j*, this correlation measure ignores any relationship region *i* may simultaneously have with regions other than *j*, potentially resulting in a loss of information [20].

To overcome this limitation, partial correlation networks, such as the sparse inverse covariance estimation with the graphical least absolute and selection operator or gLASSO [21] have become increasingly popular [22]. The gLASSO approach is particularly useful in situations where the set of observations *N* is smaller than the set of possible network connections *p* (*N* < *p* case) [20, 23]. However, in order to accommodate for this case, the gLASSO enforces sparsity in the inverse covariance estimate, and the penalised likelihood expression that needs to be optimised is not a consistent estimator [24]. While the gLASSO overcomes some the shortcomings of the PPC, it too relies on a tuning parameter, a sparsity index λ, which is often defined independent of the data and has a large effect on the resultant network. Methods to choose the optimal value of λ have been well-researched. One such method is the stability approach to regularisation selection (StARS) for high dimensional graphical models [25]. However, this approach also relies on pre-defined tuning parameters independent of the data, such as the size and number of sub-matrices to sample which is required by the algorithm [25]. For these reasons, a consistent statistical network approach that is robust to the choice of value for the tuning parameter is needed in order to deduce reliable data driven networks.

Furthermore, in an era of neuroimaging “big data”, Smith and colleagues [26] foresee the need to develop novel statistical methods, such as connectivity network estimators, which have desirable theoretical properties such as convergence to the true solution as the sample size increases (*N* > *p* case). This unmet need follows from one of the most successful and largest studies in advancing AD research, the Alzheimer’s Disease Neuroimaging Initiative (ADNI [27]) as well as several other large-scale studies [28–30] which are in the process of recruiting thousands to hundreds of thousands of participants.

### 1.1 Theoretical background of the MNL algorithm

Markov random fields (MRF) are a broad class of neighbourhood based formulations which are often included in neuroimage processing models to account for the spatial variation among voxels or ROI [31]. Conditional autoregressive (CAR) spatial models are a type of MRF which assumes a known and fixed neighbourhood adjacency structure in the form of a binary symmetric matrix *W* [32]. The covariance structure for the multivariate CAR model is a function of *W*, and, while the joint distribution of the well-known intrinsic CAR model is improper (the distribution does not integrate to one and the expected value is not defined in closed form), variations of the CAR model yield well defined multivariate distributions. For example, the Leroux et. al. (2000) [33] multivariate adaptation of the CAR model was applied by Anderson et. al. (2016) [34] in the context of aerial disease mapping. However these simple and fixed neighbourhood formulations of *W* may not adequately capture the complex spatial covariance patterns between regions of the brain.

More recently, in the context of estimating neuroimaging covariance, Cespedes et. al. (2017) [35] estimated the matrix *W* using a Bayesian hierarchical model. However, as this model consisted of 595 parameters, it was found to be too computationally intensive to estimate with Markov chain Monte Carlo methods. It is therefore desirable to develop a method to approximate *W* using a less computationally intensive approach, particularly for large data sets, as this can be very useful for exploratory purposes.

The Leroux et. al. (2000) [33] multivariate adaptation of the CAR model is a joint probability distribution of spatial observations, **b**_*i*_, conditional on the adjacency structure *W* and a spatial scale variance term, $\sigma_{s}^{2}$, which in this work will be referred to as the network likelihood. Maximum likelihood estimation (MLE) is a well-known statistical approach employed in many applications for parameter estimation [36, 37]. One of the advantages of this approach is that it only requires optimisation of the likelihood function conditional on the sample data, which is straightforward to implement in general. Furthermore, MLEs have been shown to be consistent under certain conditions [38–40], meaning that as the sample size increases, the MLE will converge with probability one to the true parameter value of the data generating process.

In this work, we propose a MLE algorithm to estimate *W* in the network likelihood, as it represents the underlying covariance connectivity brain structure, while taking into account the variation among all participants. The approach presented will henceforth be termed as maximisation of the network likelihood (MNL). Unlike gLASSO and PPC networks, the MNL returns a single binary connectivity matrix based on a consistent network estimator and is robust to the choice of value for the tuning parameter. This avoids the threshold and sparsity issues discussed earlier and provides a simultaneous analysis on the connectivity of all regions, while providing network estimates whose accuracy increases proportional to the sample size.

The layout of this manuscript is as follows. Sections 2.1 and 2.2 presents the case study used in this research. The MNL approach is described in detail in Section 2.3. The utility of this approach is then demonstrated through both a simulation study (Sections 2.4 and 3.1) and an application of cortical thickness covariance networks from structural MRI data (Section 3.3). Two network connectivity matrices are derived for groups of healthy controls (HC) and mild cognitive impaired (MCI), followed by a comprehensive discussion of the comparative merits of the MNL algorithm with the PPC and gLASSO alternatives presented in Sections 3.1 and 3.4.

## 2 Materials and methods

### 2.1 Participants of the ADNI study

The Alzheimer’s Disease Neuroimaging Initiative (ADNI) is a world wide data sharing collaboration project for AD research [27, 41]. ADNI is a multisite ongoing longitudinal study designed to assist researchers develop clinical, imaging, genetic and biochemical biomarkers for AD research. In this work, we compare cortical connectivity’s of normal healthy ageing (HC) individuals with those who have mild cognitive impairment (MCI). As cognitive impairment precedes dementia onset, individuals with MCI may include prodromal AD participants where cortical atrophy may already be present and/or in its early stages. For the current research, we used the participant’s first visit (baseline) data from 1,383 individuals; 761 male and 622 female.

Written and informed consent was obtained from all participants and/or authorised representatives and study partners. All ADNI studies are conducted according to the Good Clinical Practice guidelines, the Declaration of Helsinki, US 21CFR Part 50—Protection of Human Subjects and Part 56—Institutional Review Boards, and pursuant to the state and federal Health Insurance Portability and Accountability Act (HIPAA) regulations. Refer to http://adni.loni.usc.edu/ for full details of ADNI protocol and ethical requirements for each ADNI study.

### 2.2 Image analysis and data acquisition

In this work, we consider structural MRI scans which were undertaken at baseline. The structural MRI T1.5 and T3 weighted images were first segmented into grey/white matter and cerebral spinal fluid using an in-house implementation of the expectation maximisation algorithm applied to a Gaussian mixture model [42]. Cortical thickness was then computed along the grey matter based on the combined Lagrangian-Eulerian partial differential equations approach [43]. The automated anatomical atlas (AAL) [44] was used to parcellate the brain into 116 cortical and sub-cortical regions. In this work, we analysed 34 cortical regions from the left and right hemisphere (*K* = 68 regions total) for each individual. The remaining 48 sub-cortical regions were excluded as these analyses considers ROI cortex regions measured in *mm*, and sub-cortical ROIs such as the hippocampus are better represented by their volume rather than thickness. The anatomical regions are listed in Table 1. Once parcellated, the mean cortical thickness of the voxels in each ROI was computed and used in this analysis.

**Table 1:** List of 68 regions of interest (ROI) of the cortical mantle from the AAL. Right and left hemispheric regions correspond to odd and even numbers respectively.

Region Name	No.	Region Name	No.
Superior frontal gyrus dorsolateral	1, 2	Precentral gyrus	35, 36
Superior frontal gyrus orbital	3, 4	Supplementary motor area	37, 38
Middle frontal gyrus	5, 6	Postcentral gyrus	39, 40
Middle frontal gyrus orbital	7, 8	Superior parietal gyrus	42, 42
Inferior frontal gyrus opercular	9, 10	Inferior parietal gyrus	43, 44
Inferior frontal gyrus triangular	11, 12	Supramarginal gyrus	45, 46
Inferior frontal gyrus orbital	13, 14	Angular gyrus	47, 48
Superior frontal gyrus medial	15, 16	Precuneus	49, 50
Superior frontal gyrus orbital	17, 18	Pracentral lobule	51, 52
Gyrus rectus	19, 20	Olfactory cortex	53, 54
Anterior cingulate and paracingulate gyri	21, 22	Lingual gyrus	55, 56
Median cingulate and paracingulate gyri	23, 24	Fusiform gyrus	57, 58
Posterior cingulate gyrus	25, 26	Superior temporal gyrus	58, 60
Cuneus	27, 28	Temporal pole: superior temporal gyrus	61, 62
Superior occipital gyrus	29, 30	Middle temporal gyrus	63, 64
Middle occipital gyrus	31, 32	Temporal pole: middle temporal gyrus	65, 66
Inferior occipital gyrus	33, 34	Inferior temporal gyrus	67, 68

### 2.3 Maximisation of the network likelihood

The MNL algorithm estimates the connectivity structure via maximising the network likelihood. In this work, the network likelihood is the Leroux et. al. (2000) [33] multivariate CAR model, which is of the following form

$$\begin{array}{cc} b_{i} & ∼MVN(0,\sigma_{s}^{2}Q)\\ Q^{-1} & =\gamma (\hat{W}-W)+\left(1-\gamma \right)I, \end{array}$$ (1)

where the set of spatial observations for *K* ROIs on the *i*^*th*^ participant is **b**_*i*_ and I is the *K* by *K* identity matrix. The binary elements of the symmetric adjacency matrix *W* take values *w*_*jk*_ = 1 to denote a network link, if regions *j* and *k* have spatial similarity, or *w*_*jk*_ = 0 otherwise, which denotes the absence of a link. The diagonal elements are *w*_*jj*_ = 0 as specified by Lee (2011) [45] and Anderson et. al. (2014) [46]. Diagonal matrix $\hat{W}$ has zero off-diagonals, with the *j*^*th*^ diagonal term equal to *j*^*th*^ row sum of matrix *W*. The spatial scale variance is denoted by $\sigma_{s}^{2}$ which controls the amount of spatial variation among the *K* regions and is multiplied by the spatial covariance matrix *Q*, which is a function of *γ* and the adjacency matrix *W*. The value of *γ* represents the strength of spatial dependence on **b**_*i*_ and, in this setting, it is the tuning parameter in the MNL algorithm. Values of *γ* close to zero imply the set of spatial observations are independent and *Q* becomes a diagonal matrix. Alternatively, as *γ* approaches one, it forces *Q* to be a covariance structure with non-zero off-diagonal terms. This suggests that **b**_*i*_ has an inherent spatial covariance structure. In practice *γ* is seldom estimated and remains fixed as it is a difficult (in terms of identifiability) and computationally intensive parameter to estimate [34, 45]. In the context of brain connectivity estimation, *γ* is often set to 0.9 to enforce a relatively large spatial dependence among the observations [35]. In this work, in addition to the simulation study described in Section 2.4, we also performed a simulation study to assess the ability of the MNL algorithm (with *γ* fixed at 0.9) to recover the connectivity network on data generated on a range of *γ* values. We found that the MNL algorithm adequately recovered the simulated connectivity structure from data with various levels of spatial dependence and is hence robust to the value of *γ*, and supports our choice for fixing *γ* to 0.9. Refer to Supporting Information S1 Table for simulation results.

#### 2.3.1 MNL algorithm implementation

For *N* total participants, the likelihood function is

$$\begin{array}{cc} p(B|W,\sigma_{s}^{2}) & =\prod_{i=1}^{N}p\left(b_{i}|W,\sigma_{s}^{2}\right)\\ & =\prod_{i=1}^{N}{\left|2\pi \sigma_{s}^{2}Q\right|}^{-\frac{1}{2}}\exp(-\frac{1}{2\sigma_{s}^{2}}b_{i}^{T}Q^{-1}b_{i}). \end{array}$$ (2)

Maximisation of Eq (2) is performed using 15 steps as shown in Algorithm 1. The MNL algorithm provides iterative updates on W* and $\sigma_{s}^{2*}$ *M* times. The fast quasi-Newton algorithm implemented to update $\sigma_{s}^{2*}$ was adapted from Byrd et. al. (1995) [47]. As this is a deterministic algorithm, Step 14 of Algorithm 1 repeats the search for *W* for *P* sets of different starting values to mitigate being stuck in a local minima.

**Algorithm 1:** MNL algorithm

**Input:** Set of spatial observations *B*, random binary matrix W* and small positive spatial variance $\sigma_{s}^{2*}$

**Output:** *W* and $\sigma_{s}^{2}$ estimates that maximise the network likelihood

**1** Evaluate log-likelihood $\delta^{*}=\log\left[p\left(B|W^{*},\sigma_{s}^{2*}\right)\right]$

**2** **for** *M runs* **do**

**3**  **foreach** w element of W* **do**

**4**   Permute *w*^*th*^ element in W* to get W**

**5** $\delta^{w}=\log\left[p\left(B|W^{**},\sigma_{s}^{2*}\right)\right]$

**6**   **if** *δ*^*w*^ > δ* **then**

**7**    δ* = *δ*^*w*^

**8**    W* = W**

**9**   **end**

**10**  **end**

**11**  Update $\sigma_{s}^{2*}$ conditional on W* with a fast quasi-Newton algorithm

**12** **end**

**13** Retain final W* and $\sigma_{s}^{2*}$

**14** Repeat Steps **1** to **13** at different random starting values *P* times

**15** Return W* and $\sigma_{s}^{2*}$ estimates corresponding to the highest δ*

#### 2.3.2 Data processing

To estimate the model proposed in Eq (1) based on real data, a linear regression was applied to the set of ROI observations **y**_.*k*_ = [*y*_1*k*_, *y*_2*k*_, …, *y*_*ik*_, …, *y*_*Ik*_] for each ROI over all *I* individuals. Covariates in these linear regression models included gender, apolipoprotein (APOE) *ε*4 carrier and non-carriers status and age in a similar manner as previous studies [8, 15, 17]. The predicted values of the regression model $\left({\hat{y}}_{ik}\right)$ were obtained, and the residuals for each set of ROIs were computed by $\varepsilon_{ik}={\hat{y}}_{ik}-y_{ik}$. These residuals were standardised by $b_{ik}=\left(\varepsilon_{ik}-{\bar{\varepsilon }}_{k}\right)/s_{k}$, where ${\bar{\varepsilon }}_{k}$ and *s*_*k*_ are the empirical mean and standard deviation of the residual for each region over all individuals. The MNL algorithm was applied to the final set of observations (*b*_*ik*_). This transformation allows for the residuals of the ROIs to be on the same scale, (as the variance of each *ε*_.*k*_ is set to one) while maintaining the correlation structure of the data after accounting for covariates. Pairwise plots and histograms of the transformed residual showed linear relationships between certain ROIs and each ROI were approximately Normally distributed and centred at zero (not shown).

### 2.4 Simulation study of MNL algorithm

The goal of the simulation study is to assess the ability of the MNL algorithm described in Section 2.3 to recover binary connectivity matrices based on simulated neural data at various sample sizes. A further assessment focused on comparing the results of the MNL algorithm with those obtained using the gLASSO and PPC methods applied to the same simulated data.

**Fig 1:**
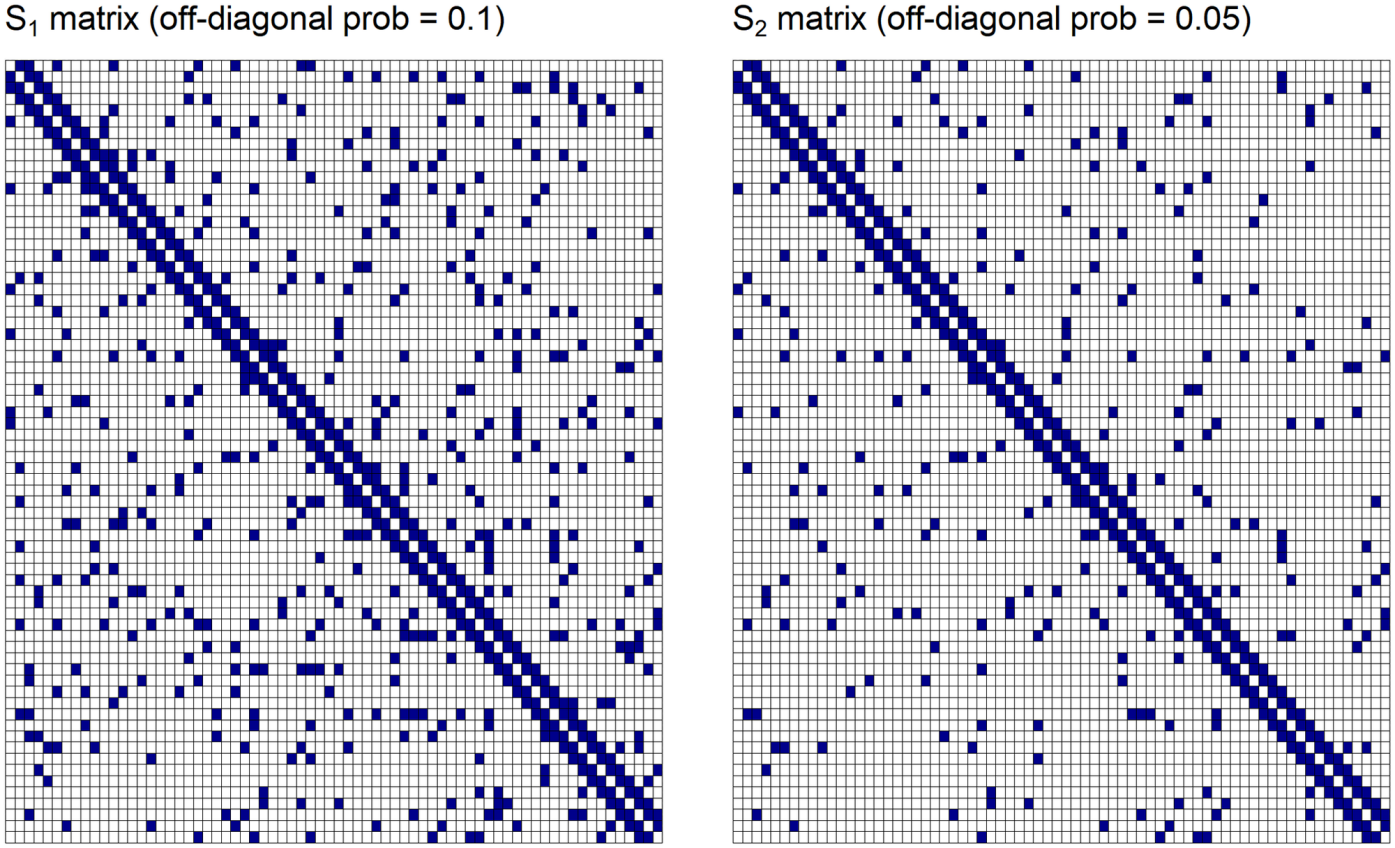
Simulated networks *S*_1_ and *S*_2_. *W* connectivity matrices to recover in simulation study. Semi-sparse (left) and sparse (right) second order random networks denoted as *S*_1_ and *S*_2_ respectively. Off-diagonal elements had a probability of 0.1 and 0.05 of a link present (in blue) and 0.9 and 0.95 probability of an absent link (cells in white).

Our simulation study comprised of two simulated networks, *S*_1_ and *S*_2_ as shown in Fig 1. We combined a second order diagonal network with a random network model as described in Bien and Tibshirani (2011) [48]. As cortical networks, in general, have a diagonal structure [14], both binary solution networks had second order connections *S*_*i*,*i*−1_ = *S*_*i*−1,*i*_ and *S*_*i*,*i*−2_ = *S*_*i*−2,*i*_ = 1 and zero otherwise. A random network model was used to simulate semi-sparse (*S*_1_) and sparse (*S*_2_) off-diagonal elements, whereby the remaining off-diagonal elements had a probability of 0.1 and 0.05 respectively, of a connection being present. To convert these binary solution networks into covariance matrices, in a similar manner as Bien and Tibshirani (2011) [48], the diagonals and off-diagonal elements of *S*_1_ and *S*_2_ were multiplied by different positive constants resulting in covariance matrices Ω_1_ and Ω_2_. Data were generated from a multivariate normal distribution *MVN*(**0**, Ω) for each covariance matrix for sample sizes *N* = 100, 250, 500, 1000. Ten independent sets of simulated data were drawn for each sample size in order to allow for a rigorous comparison of performance of each method at every sample size.

To assess the performance of the MNL algorithm we compared the rate of the true positive connections (sensitivity), which was summarised by the percentage of the connections which were correctly identified to be present. Likewise, the true negative rate (specificity) was summarised by the proportion of absent connections that were correctly identified by the algorithm. As the connectivity matrices are symmetric, we only consider the upper off-diagonal elements of each matrix. A network classifier which has a perfect recovery of the solution network will have both sensitivity and specificity percentages close to one. Alternatively, a poor performing algorithm will have the respective percentages close to zero.

Comparison of the performance of the MNL, gLASSO and PPC methods for increasing sample sizes provides insight into the consistency of each approach. A consistent network estimator has a property that as the sample size increases, the estimated network converges to the true solution [37]. Mathematically, we can demonstrate that the parametrisation of $1/\sigma_{s}^{2}Q^{-1}$, and by extension $\sigma_{s}^{2}Q$, is a positive definite covariance matrix for all values of *γ*, refer to Supporting Information S1 File: *Proos MNL is a consistent estimator* for a proof of this result. It follows from fundamental theoretical results by Greene 2010 (Chapter 14 [49]) and Pourahmadi (2000) [50] among others, that an MLE estimator of a positive definite covariance matrix is a consistent estimator, and will converge to the true solution with probability one as the sample size increases.

In addition to assessing the MNL algorithm as a suitable candidate for network estimation, the simulation study also provides information on the performance of each algorithm according to different sample sizes based on two network configurations for a range of tuning parameters (for gLASSO and PPC methods).

### 2.5 Alternative brain connectivity methods

As described in Section 1, the PPC and gLASSO are current and popular methods used to derive connectivity networks. In this section we provide a brief description of each approach in relation to the simulation study and its application to the case study.

#### 2.5.1 PPC approach

The PPC continues to be a popular approach to derive cortical connectivity networks [19] and, for this reason, this approach will also be considered in our simulation study. The correlation between region *i* and *j* is denoted by *ρ*_*ij*_ and −1 ≤ *ρ*_*ij*_ ≤ 1. All possible sets of pairwise correlations (*ρ*_*ij*_) were used to populate the correlation matrix [2, 8, 15, 51, 52]. A binary adjacency matrix *A* was derived from the correlation matrices, with elements *a*_*ij*_ equal to zero if |*ρ*_*ij*_| < *τ* and value one if |*ρ*_*ij*_| ≥ *τ*, where the threshold range or tuning parameter is 0 < *τ* < 1, similar to the threshold range described by He et. al. (2008) [51]. Fewer spurious correlations are included as *τ* approaches one, and this may result in disconnected networks determined by the strongest correlations. Alternatively, if *τ* is too close to zero then the highly connected network may include connections which arose due to spurious noise from the data. In practice, the threshold range in cortical correlation analyses is chosen such that the resultant networks have several organisational features such as small-world topology and the minimum clustering coefficient is above zero in order to make meaningful comparisons between networks [6, 15, 51, 53]. In contrast, the estimated *W* from the MNL algorithm does not rely on tuning parameters and organisational network features described above can be evaluated directly on this estimated network. In this work, values for *τ* in {0.1, 0.15, 0.2, 0.25, …, 0.75, 0.8, 0.85} were initially investigated for the PPC networks, and this was fine-tuned for the simulation study.

#### 2.5.2 gLASSO algorithm

The graphical LASSO is a fast approach to estimate a sparse inverse covariance matrix [21, 22, 54]. For a set of observations **b**_.1_, **b**_.2_, …, **b**_.*I*_ from a multivariate normal distribution **b**_.*i*_ ∼ *MVN*(**0**, Γ) with precision matrix Θ = Γ^−1^, gLASSO aims to find $\hat{\Theta }$ such that

$$\begin{array}{c} \hat{\Theta }=\text{max}\{\text{log}\left(|\Theta |\right)-\text{trace}\left(S\Theta \right)-{\lambda ∥\Theta ∥}_{1}\}, \end{array}$$ (3)

where the sample covariance matrix is denoted by *S* and ||.||_1_ is the *L*_1_ norm. The sparsity tuning parameter λ, also known as the penalizing parameter, determines the sparsity of $\hat{\Theta }$. For example, high values of λ implies that ||Θ||_1_ has a large contribution to the optimisation problem in (3). Conversely, when λ = 0, Eq (3) reverts to a simpler MLE problem.

In terms of brain connectivity, the gLASSO is applied to estimate Θ, which is then used to derive the binary connectivity matrix. Values of this network matrix are equal to one if the corresponding values of $\hat{\Theta }$ are non-zero, and an absent connection is defined by the zero values of $\hat{\Theta }$. Brain connectivity networks estimated by (3) include the work by Huang et. al. (2010) [20] and Cho et. al. (2017) [23] among others [55, 56]. Authors Huang et. al. (2010) [20] focused on the investigation of network organisation and selected λ such that the networks had a fixed number of links. Alternatively, the StARS approach was used to derive an optimal value for the tuning parameter λ in Cho et. al. (2017) [23] and the effect of λ on the results were not investigated. In addition to the StARS approach, extensive research and development in relation to the optimal λ value has led to several novel approaches, including cross validation [24] among others [57], refer to Fan and Feng 2009 [58] for a review.

In this work, we are interested on the effect λ has on the performance of gLASSO and its ability to correctly identify the solution networks for *S*_1_ and *S*_2_. Values for λ in {0.1, 0.15, …, 1.7} were explored for gLASSO networks in the simulation study, and a subset of this range was chosen for the real data analyses. Furthermore, we also explored the optimal value of λ which minimises the cross validation error, as this is one of many standard approaches to derive the value of λ [24] (See Supporting Information S3 Fig for results). As the intention of this research is to present the MNL algorithm, further investigation of networks derived by gLASSO with other approaches to derive λ is beyond the scope of this work.

### 2.6 Statistical analysis

Via exploratory data analyses, the demographic characteristics were compared between HC and MCI participants over age using an Analysis of Variance, Independent Sample t-test, Chi-squared tests (gender and APOE *ε*4 allele positive status), as well as Kruskal Wallis test (MMSE and CDR). All statistical analyses were performed using the R statistical environment (R version 3.4.2, R Core Team [59]).

On both simulation and real data application, a single application of the MNL algorithm took less than a minute to run on a single central processing unit (CPU) on a standard computer (four core 3.40GHz Intel i7-4770 processor). We expect this to vary for different *N* and *K* data sets. Step 5 of Algorithm 1 is executed in C^++^ in order to improve the computational time taken to run the MNL algorithm. The remainder of the algorithm is implemented in R. We note that nested for-loops (Steps 2 and 3 of Algorithm 1) act as a bottle neck and future work to profile Algorithm 1 would speed up the implementation of the MNL algorithm. In this work, the MNL algorithm was found to be slightly slower (in terms of seconds) than the gLASSO and PPC. Refer to https://github.com/MarcelaCespedes/MNL_algorithm for the coded implementation of the MNL algorithm, full simulation study as well as a tutorial on the implementation of the MNL approach.

## 3 Results

### 3.1 Simulation study: Comparison of MNL, gLASSO and PPC algorithms

The aim of this simulation study was to evaluate the performance of the MNL algorithm to correctly recover the connectivity matrices for each configuration shown in Fig 1 from the simulated data described in Section 2.4. As the simulation study described in Section 2.3 shows that for a range of *γ* the performance of the MNL algorithm is relatively robust in terms of the the sensitivity and specificity of the recovered network, in this simulation study we assess the performance of the MNL algorithm against a range of threshold and sparsity values for the PPC and gLASSO.

#### 3.1.1 Simulated semi-sparse network *S*_1_

**Fig 2:**
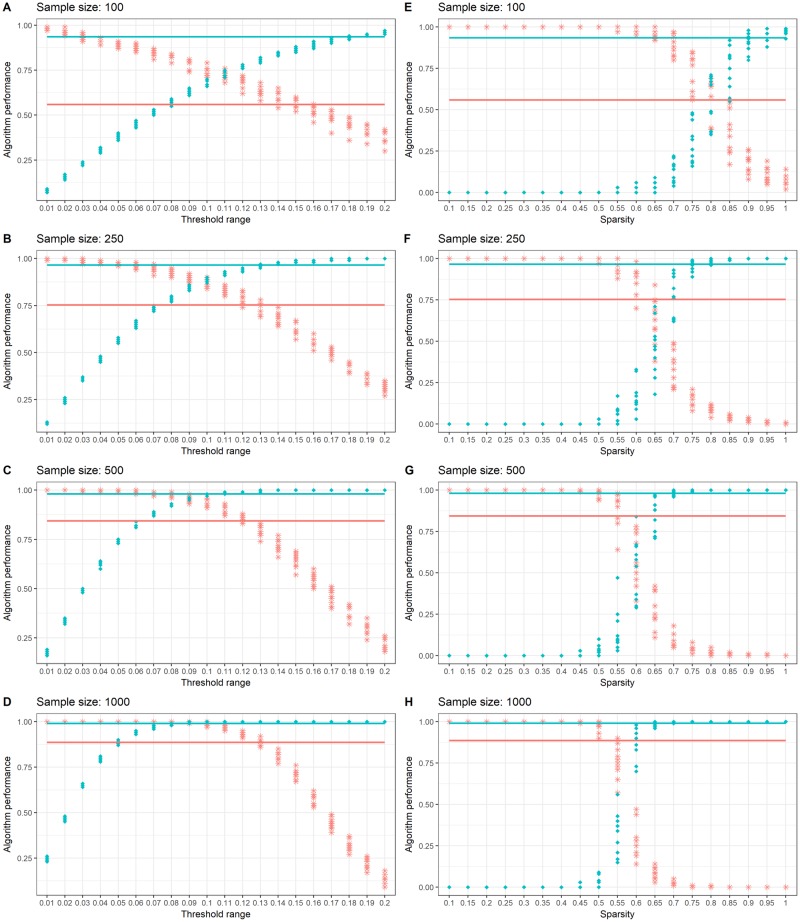
Simulation results for *S*_1_. Sensitivity (in red stars) and specificity (in blue dots) for *S*_1_ simulation results for PPC (left column: A, B, C and D) and gLASSO (right column: D, F, G and H). Simulation results highlight the effect of threshold and sparsity tuning parameters for the PPC and gLASSO algorithms over sample sizes *N* = 100, 250, 500 and 1000. MNL algorithm sensitivity and specificity results are shown by the horizontal lines which are the average over all 10 replicates for each sample size, colour coded in red and blue respectively.

As described in Section 2.4, out of the two simulated matrices considered in this work, *S*_1_ is the semi-sparse diagonal network. Each sample size comprised of ten replicates and Supporting Information S2 Fig shows the covariance plots for randomly selected covariance matrices for each sample size. Fig 2 shows the gLASSO and PPC results and their ability to correctly identify the elements of the *S*_1_ matrix via the sensitivity and specificity for all simulated data over a range of tuning parameters.

The sensitivity and specificity of the MNL algorithm are shown by the red and blue horizontal lines respectively, refer to Table 2 for values. Supporting our theoretical results which show that the MNL is a consistent estimator, our simulation study shows that as the sample size increases, both sensitivity and specificity approach one. It is interesting to note that for all sample sizes the MNL algorithm in general has a specificity close to one, suggesting that the algorithm has a high chance of detecting no link when no such link exists. The simulation study in this work show that the MNL algorithm is better suited for applications where it is desirable to avoid over-interpreting incorrect links. While this trait has it obvious merits, the MNL algorithm may be unsuitable in applications such as gene regulatory networks, where it is desirable to over-select the network connections rather then underestimate them [25].

**Table 2:** MNL simulation results. MNL simulation study results across sample size (*N*) for *S*_1_ and *S*_2_ simulated networks. Sensitivity and specificity values averaged over ten replicates.

*N*	*S*_1_		*S*_2_	
	Sensitivity	Specificity	Sensitivity	Specificity
100	0.56	0.94	0.51	0.94
250	0.75	0.97	0.73	0.96
500	0.84	0.98	0.85	0.97
1000	0.89	0.99	0.91	0.98

Both gLASSO and PPC approaches show a trade-off between the ability to correctly detect the presence and absence of links over the range of values of *τ* and λ. In general, for all sample sizes and small tuning parameters, both algorithms show a sensitivity close to one but a specificity close to zero, suggesting that these algorithms largely overestimated the number of links of the networks. At the other extreme of the tuning parameters, this relationship switches, and resultant networks approach a zero connectivity matrix reflected by specificity close to one and a sensitivity close to zero. Our results illustrate the extent the tuning parameters can have on the gLASSO and PPC, making the correct choice in practice difficult; as the optimal performance of the gLASSO and PPC occurs when the specificity and sensitivity are at their highest and this occurs within a very narrow range of the tuning parameters. This simulation study also serves to show the benefits of an approach that is robust to the choice of value for the tuning parameter, as the MNL algorithm is not affected by such trade-off.

In relation to the PPC, the value of *τ* can range between zero and one, however, a smaller range away from the extremes is often used in practice to avoid the issues described in Section 2.5.1. It is surprising to see in our simulation study that at small values of *τ* = 0.08, 0.09, 0.1 and 0.11, the PPC offers superior performance compared to the other two alternatives, particularly at a sample size of *N* = 1000. While it is unlikely that these values of *τ* are used in practice, we note that the approach to simulate the data favours the PPC approach. As the sample covariance matrices in Supporting Information S2 Fig show a clear difference between covariance values for present and absent links. This difference is mostly emphasised as the sample size increases to *N* = 1000.

Across all sample sizes, the optimal performance of the gLASSO occurs at sparsity values λ = 0.55, 0.6, 0.65 and 0.8 as shown in Fig 2. Outside of these values, gLASSO shows the trade-off between sensitivity and specificity giving poorer performance. In comparison to the MNL algorithm, particularly at λ values where gLASSO had optimal performance, the MNL algorithm maintained similar or superior specificity and sensitivity across all sample sizes. As the MNL algorithm relies on the evaluation of the full likelihood, its application is not always suitable for small sample sizes (*N* < *p* case). As suggested in literature the gLASSO may be better suited for brain connectivity estimation in smaller clinical studies [20]. Our simulation study support this result in the case of *N* = 100 and *K* = 68 ROIs, as the gLASSO results were comparable to those those from the MNL algorithm. We note that unlike the bounded tuning parameters of the PPC, the range λ can take are all positive values, making the choice of λ in practice more difficult to ascertain than the PPC approach.

#### 3.1.2 Simulated highly sparse network *S*_2_

The data generating network for *S*_2_ has approximately half the connections than the *S*_1_ network and for this reason it is of interest to see how the MNL and PPC algorithm perform, and in particular how they compare to the gLASSO which is specifically designed to estimate sparse networks. Refer to Supporting Information S2 Fig for covariance plots for data generated by the *S*_2_ binary networks.

**Fig 3:**
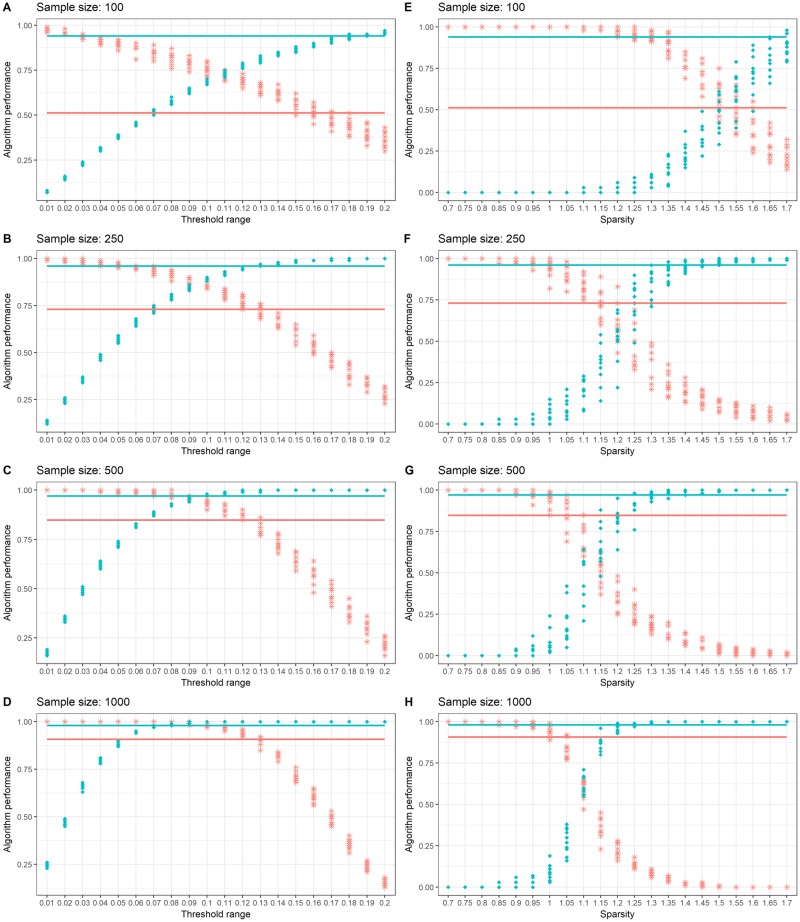
Simulation results for *S*_2_. Sensitivity (in red stars) and specificity (in blue dots) for *S*_2_ simulation results for PPC (left column: A, B, C and D) and gLASSO (right column: D, F, G and H). Simulation results highlight the effect of threshold and sparsity tuning parameters for the PPC and gLASSO algorithms over sample sizes *N* = 100, 250, 500 and 1000. MNL algorithm sensitivity and specificity results are shown by the horizontal lines, colour coded in red and blue respectively.

Simulation results in Fig 3 follow similar trends as those described in Section 3.1.1 for the gLASSO and PPC. The trade-off between sensitivity and specificity across the tuning parameters remains and the PPC demonstrates superior performance over the MNL and gLASSO algorithm at similar *τ* values as described above, particularly at a sample size of *N* = 1000. In this scenario, there is a faster improvement of the MNL algorithm as the sample size increases and we believe this is due to the simulated covariance values are larger for *S*_2_ than *S*_1_, and this is reflected in their respective covariance plots. Refer to Table 2 for MNL algorithm results.

As the connectivity matrix we are interested in recovering is a highly sparse network in comparison to *S*_1_, it is not surprising to see that the performance of the gLASSO also improves faster as the sample size increases. In this simulated scenario, optimal performance of the gLASSO occurs at λ = 1.55 on a sample size of *N* = 100 and this improves for an optimal sensitivity and specificity of 0.62 with λ = 1.1 at a sample size of *N* = 1000. The MNL algorithm shows higher sensitivity and specificity values compared to the gLASSO at sample sizes greater than 250. It is interesting to note, that in this simulation study the optimal values of *τ* remain, in general, unchanged for the PPC approach, whereas for the gLASSO, there is a large difference in the range of λ for *S*_1_ and *S*_2_ matrices where optimal performance occurs.

### 3.2 Case study: Characteristics of study participants

The results of the exploratory data analyses of the demographic features of the participants in the study are shown in Table 3. A chi-squared test for independence found a significant association between gender and diagnosis levels (*p* < 0.0001), as the MCI group had a considerably higher number of males compared to the HC group. Compared with HC, MCI participants were more likely to have the variant APOE *ε*4 allele (*p* < 0.0001). Cortical thickness measures for all 68 ROI were significantly higher in HC participants (mean 2.71 *mm*) compared with MCI participants (mean 2.66 *mm*) (*p* < 0.0001). Significant ordinal patterns of degeneration from HC to MCI were observed as follows: cognitive Mini Mental State Examination (MMSE) scores decreased from 29 to 28 (*p* < 0.0001); Clinical Dementia Rating (CDR) score values increased from 0 to 1.5 (*p* < 0.0001). As each individual has 68 ROI observations, here, the smallest sample size comprises of 35,156 observations which is greater than 2,278 potential links for a 68 × 68 connectivity matrix.

**Table 3:** Summary of ADNI case study data. Subset of ADNI cohort demographic characteristics for individuals studied at baseline. Age mean reported with standard deviation in parenthesis. Mini-mental state exam (MMSE) and clinical dementia rating sum of boxes (CDR-SOB) medians reported with inter-quartile range in parenthesis. Genetic assessment included the apolipoprotein (APOE) *ε*4 carrier and non-carrier status. Data included missing APOE *ε*4 status for a single HC and three MCI individuals.

N = 1,383 participants			
	HC	MCI	p-value
Total no.	517	866	
Age	74.27 (5.78)	73.05 (7.60)	0.00073
Female	268 (52%)	354 (41%)	0.0001<
APOE *ε*4 carriers	147 (28%)	435 (50%)	0.0001<
MMSE	29 (1)	28 (3)	0.0001<
CDR	0 (0)	1.5 (1)	0.0001<

### 3.3 Case study: MNL analysis

Prior to applying the MNL algorithm, we observed the histograms of the **b**_.*i*_ values for each region. These plots confirmed that the transformed data follow an approximate Normal distribution centred at zero (plots not included).

**Fig 4:**
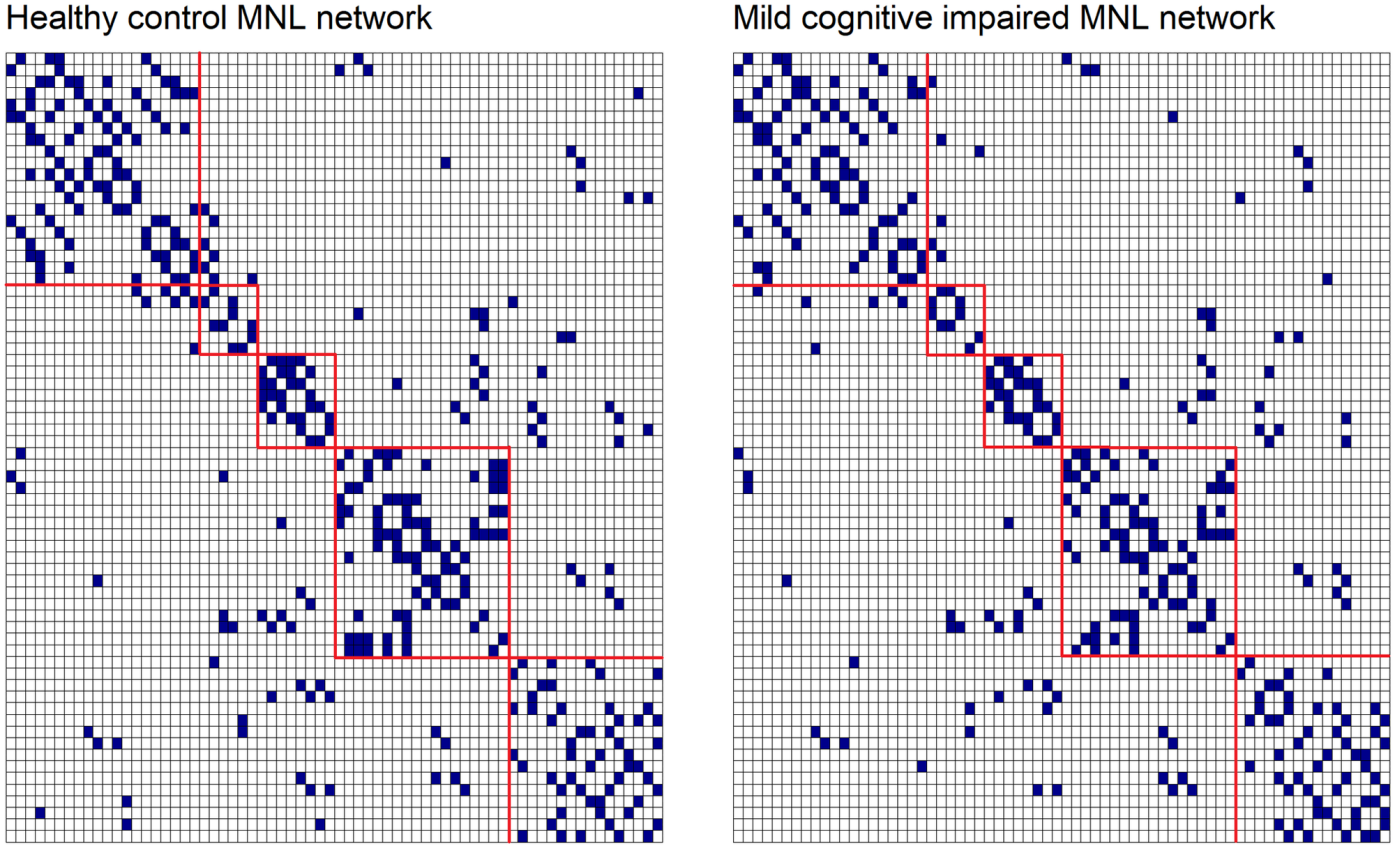
MNL networks on case study data. MNL connectivity networks for HC (left) and MCI (right) diagnosis. Blue denotes a link between two ROIs and white denotes absence of connections, cerebral frontal, limbic, occipital, parietal and temporal lobes outlined in red. Refer to Table 1 for ROI names.

Inspection of pairwise plots of the transformed data showed the association between regions displayed various levels of linear relationships, suggesting there is a covariance structure in the data (plots not included). Representative samples from these plots, such as paired regions 23 and 49, 48 and 60, 42 and 52 suggest there is a linear relationship among these regions for all diagnosis groups, refer to Table 1 for ROI names and Supporting Information S3 Fig for plots. Hence, the covariance structure of the MNL algorithm in Fig 4 show these regions to be connected. Likewise, the absence of a linear relationships was observed in pairwise plots between, for example, regions 21 and 30, 36 and 57, 51 and 68, across both diagnosis groups. The lack of association between these regions is indicated by the corresponding absence of links in the networks of Fig 4. Furthermore, as a goodness-of-fit assessment of the MNL algorithm, we examined the residuals after we fitted the model to the transformed data from the two diagnosis groups. Histograms and scatter plots of the residuals show they were approximately Normally distributed, refer to Supporting Information S5 Fig.

In order to assess if the MNL algorithm adequately modelled the spatial structure of the data, we computed the Moran’s I statistic [60] on the set of residuals from the MNL model fitted to the data for each person within each diagnosis group [61, 62]. Synonymous to Pearson’s correlation, a Moran’s I value close to zero, contingent on spatial structure matrix *W*, indicates the data have low spatial correlation [63]. The median Moran’s I value for HC and MCI groups were found to be equal to or less than 0.31. Correlation and partial correlation plots of the MNL algorithm residuals in general had values which were substantially small, refer to the Supporting Information S5 Fig for plots. In summary, the selected pairwise, partial correlation plots and Moran’s I values suggest the covariance structure of the data on all diagnosis groups was adequately modelled by expression (2), and the histograms support the Normality assumption in (2).

The binary matrices from the MNL algorithm applied to the case study data are shown in Fig 4. These matrices represent the estimated general connectivity structures for the HC and MCI diagnosis groups. The total number of potential connections on a 68 ROI network is 2,278. The total number of links in the diagnosis networks are 180 and 167 for HC and MCI networks, respectively. The networks in Fig 4 shows a large overlap in connectivity between the networks, with 136 connections in common. Most of the connections which are common to both HC and MCI groups include those within each lobe, while most of the differences tend to occur between lobe connectivity. While there was only a subtle reduction in connectivity along the diagnosis spectrum (from HC to MCI), all estimated networks were connected, suggesting that, at some level, all ROIs co-vary with each other in that there were no regions independent from the rest.

Our initial investigation was performed on a clinical study with smaller sample sizes (HC: 171, MCI: 46 and AD: 29). However, based on the simulation study results in Section 3.1, it is clear that the performance of the MNL algorithm improves as the sample size increases. Hence in this work we applied our method on the ADNI case study on two large groups (HC and MCI), with expected pathological differences in connectivity.

From the networks in Fig 4, additional network analysis can be applied to the network matrices to determine small-world topology [4] and organisational network features such as characteristic path length and clustering coefficient [64], however, this is beyond the scope of the present study. The results from simulation studies in Section 3.1 suggest that the obtained networks are relatively reliable, and as the sample size increases, the performance of the MNL algorithm improves in both the ability to correctly identify the presence and absence of connections.

### 3.4 Case study: gLASSO approach

Our intention of applying a competing algorithm to the case study is to compare a single binary network between the MNL algorithm to a current known approach. There are several alternatives available for estimating such a network via the gLASSO, and for this reason we applied the gLASSO to the case study data. As we have yet to find methods to choose a single value for *τ* given the data, in this application we did not apply the PPC to the case study data.

**Fig 5:**
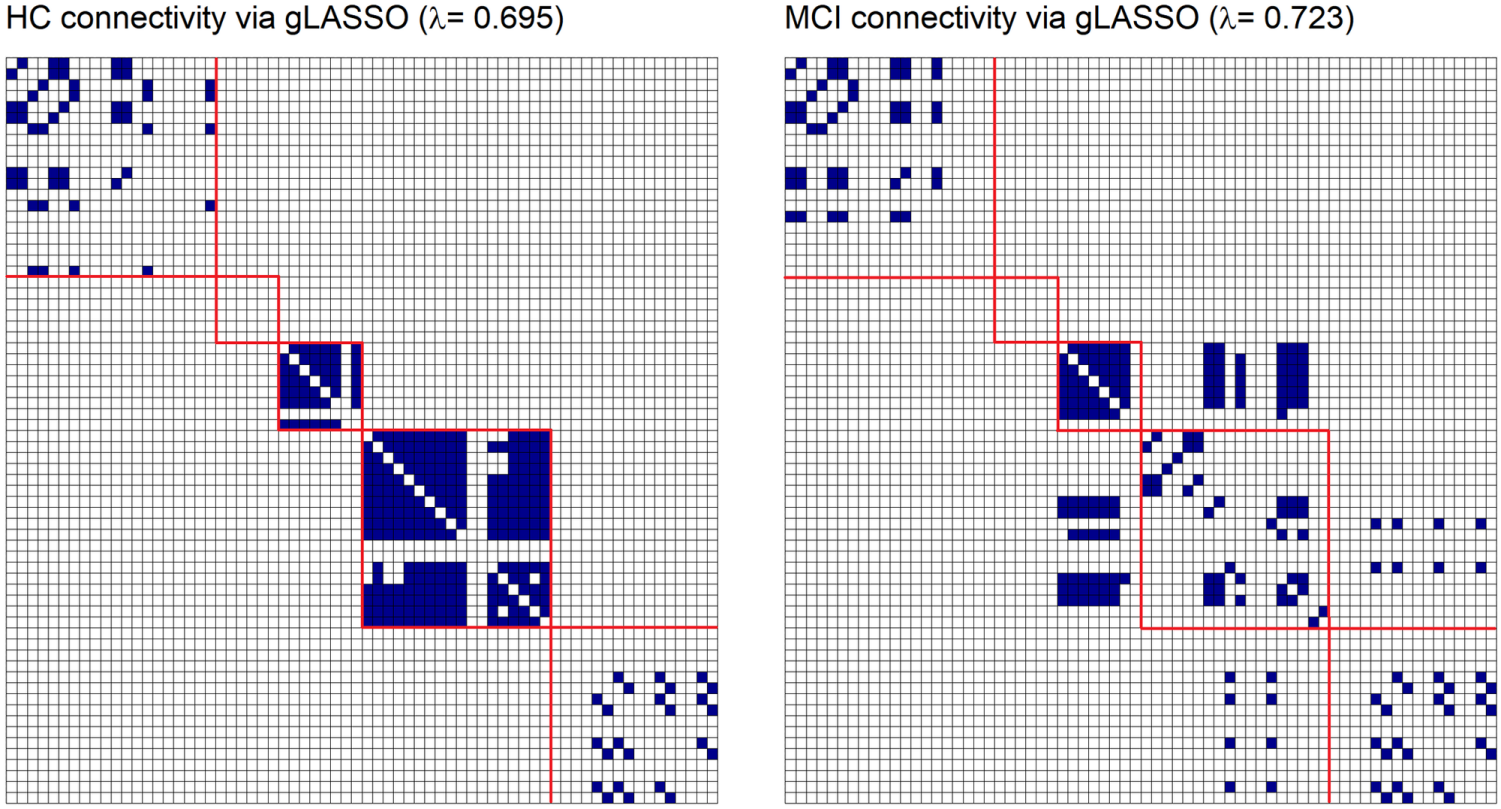
gLASSO networks on case study data. gLASSO connectivity structures for HC (left) and MCI (right) diagnosis. Blue denotes a link between two ROIs and white denotes absence of connections, cerebral frontal, limbic, occipital, parietal and temporal lobes outlined in red. Refer to Table 1 for ROI names.

Fig 5 shows the connectivity matrices for selected sparsity (λ) values, whose total links were similar to the MNL results. Without knowing the correct value of λ we first applied the glASSO for the range of sparsity values λ = {0.1, 0.15, …, 1} to understand the effect λ had on estimated networks. The resultant networks ranged between 2,278 to 0 in total number of links, refer to Supporting Information S2 Table for full results.

In a similar manner as Huang and colleagues (2010) [20], the sparsity value was chosen such that the resultant networks had a similar number of links to those from the MNL algorithm in Section 3.3. We note that the primary intention of applying the gLASSO to the case study data is to compare the change in connectivity, with less focus on finding the best model fit. Fig 5 shows the resultant networks for HC and MCI groups and the total number of links for each network were 171 and 122 for HC and MCI groups respectively.

The networks in Fig 5 show clearer block diagonal matrices, in comparison to the MNL algorithm (Fig 4), suggesting that in this work, gLASSO networks detected higher inter-lobe connections rather than between lobe connections. No connections were detected within or between the limbic lobe. In a similar manner as the MNL algorithm, there was a large overlap between the connectivity within each lobe for the HC and MCI groups, with 66 links in common. The HC network is shown to have no connections between the parietal and occipital lobes, however, in the MCI network there is a large change in connections between the occipital and temporal lobes. We note that in order to appropriately assess the network organisation (by investigating the clustering coefficient, efficiency and small world topology of the networks) and thereby further discuss biological and neurological differences between the MNL and gLASSO networks, a suitable range of λ is required, and this is beyond the scope of this work.

## 4 Discussion

In this work, we propose a novel approach to estimate brain networks from neuroimaging data. Validated on a numerical simulation study, the sensitivity and specificity performance of the MNL algorithm was shown to improve as the sample size increases supporting our theoretical results that the MNL algorithm is a consistent network estimator. In the simulation study for sample sizes greater than 100, the MNL algorithm was shown to have a higher sensitivity and specificity compared to the results from gLASSO, over a range of sparsity values. At the range of 0.08 ≤ *τ* ≤ 0.11, the PPC was shown to outperform both MNL and gLASSO algorithms, particularly at a sample size of *N* = 1000. Application of the MNL algorithm to the ADNI case study identified a loss of connections between HC and MCI connectivity networks, suggesting evidence of atrophy along the neurodegeneration pathway, supporting biologically meaningful results.

Our simulation studies found that the PPC and gLASSO analyses were sensitive to the tuning parameters in terms of the ability to recover the solution networks. A trade-off exists between the specificity and sensitivity rates in all sample sizes considered in this work, which showed that as the tuning parameters (threshold *τ* and sparsity λ) increase the specificity increases, but the sensitivity decreases and vice versa. Application of the MNL approach yields a single connectivity structure that is robust to the value of the tuning parameter (*γ*) which serves as a descriptive network statistic which is beneficial for exploratory purposes. As such, interpretation of the resultant network is limited to the specific sample used to derive the network. The brain wombling models applied to neuroimaging data by Cespedes et. al. (2017) [35] utilise expression (2) as part of a Bayesian wombling model to estimate the network connectivity and its associated uncertainty. To compare our exploratory approach with those from the Bayesian wombling models, we applied the MNL algorithm to HC, MCI and AD diagnosis groups and 35 ROIs selected in the work by Cespedes et. al. (2017) [35], albeit to baseline data only. We found that the MNL diagnosis networks correctly identified over 83% of the links (correctly detected the presence and absence of connections) obtained in the wombling model (see Supporting Information S6 Fig), suggesting the MNL algorithm can provide results that are comparable to those of Bayesian probabilistic network models.

### 4.1 Extensions

Despite the substantive appeal of the MNL algorithm described in this paper, there are several extensions that could be considered. Firstly, the current mean of the multivariate network distribution is zero and as such the MNL algorithm does not provide ROI mean estimates. Extending the MNL algorithm to include a non-zero region mean vector ***μ*** may be informative as not all ROIs have the same mean. In this work we compensated for this by applying linear regression models to each ROI and transforming the residuals such that they have a mean of zero (Section 2.3.2). We note that the added complexity of the proposed extensions to the MNL approach may result in a more difficult optimisation problem and may require more sophisticated numerical optimisation methods to estimate the additional parameters. Secondly, analyses on longitudinal neuroimaging studies are favoured in contrast with cross sectional analyses, as they could potentially include information on ROI changes over time [65]. While the MNL algorithm presented in this work does not account for repeated measures, an extension of expression (1) to account for repeated measures can be achieved by adding a random effects layer in the model. However, as the MNL algorithm is the first brain network algorithm of its kind whose connectivity estimates improves as the sample size increases, such an extension is left as future work.

### 4.2 Conclusion

The potential application of MNL networks is not restricted to cortical thickness structural MRI data, and can easily be applied to any complete spatial set of observations from any neuroimaging modality. The objective for the methodology and application presented here is to introduce and demonstrate the utility of the MNL algorithm, as the application of MNL method can be applied to functional MRI, positron emission topography (PET) and electroencephalography (EEG) data. Other than the suggestions already discussed, an additional area for future work is the application of the MNL algorithm to assess for network robustness as described in Bernhardt et. al. (2011) [16] and Hart et. al. (2016) [11]. Here, the authors investigate the loss of random or targeted nodes or edges removed from the network, representing deterioration due to pathology. Furthermore, additional validation of the MNL algorithm on other neurological applications such as epilepsy [16] and schizophrenia [15, 66], as well as healthy ageing studies over a wide age range, and analyses of network topological metrics [8] are needed to better understand the performance and biological insight from the proposed MNL algorithm.

## Supporting information

S1 TableSensitivity and specificity results for data generated with various λ values for *S*_1_ connectivity matrix.A further simulation study was performed in order to assess the performance of the MNL algorithm (with *γ* set to 0.9) applied to simulated data with various levels of spatial dependence. Simulated data from the model (1) was generated with *γ* values {0.60, 0.70, 0.80, 0.90, 0.99}, $\sigma_{s}^{2}=1$ and *W* = *S*_1_ as shown in Fig 1 of the manuscript. The sample size was set to *N* = 500.(PDF)

S2 Table Total number of links from gLASSO network applied to ADNI data. HC and MCI total number of network links derived by gLASSO for the range of 0.1 ≤ λ ≤ 1. This range encompasses a full networks where each ROI is connected with each other at 2,278 links (λ = 0.1), to the null networks with no connections (λ = 1).
(PDF)

S1 File Proof MNL is a consistent estimator. (PDF)

S1 Fig CV error in search for optimal λ. Cross validation mean and error intervals on simulated study for data simulated from binary matrices *S*_1_ (top) and *S*_2_ (bottom) for all ten replicates. Vertical black lines denote the optimal specificity and sensitivity λ trade-off as per manuscript Figs 2 and 3. Optimal value of λ is chosen such that the CV error is at its lowest.
(PDF)

S2 Fig Sample covariance matrix for *S*_1_ (top) and *S*_2_ (bottom). Sample covariance matrices for various sample sizes generated by *S*_1_ semi-sparse and *S*_2_ sparse matrix.
(PDF)

S3 Fig Selected ROI pairs which support the presence and absence of connections via the MNL. Pairwise scatter plots for selected HC and MCI ROIs. Top: ROI pairs denote linear association and correspond to link in estimated networks in Fig 4 of manuscript. Bottom: selected ROI pairs denoting an absent linear association and correspond to absent connections in Fig 4 of the manuscript.
(PDF)

S4 Fig Residual scatter plot and histogram of the residuals from the MNL algorithm applied to the ADNI case study data. Top: residual scatter plots for HC (left) and MCI (right). Bottom: Residual histograms for HC (left) and MCI (right). As these residuals are approximately normal and the scatter plot shows no apparent deviations from zero, we conclude that there were no violations on our MNL model assumptions. Furthermore, we applied Moran’s I to each set of residuals on both HC and MCI groups to assess if there was still spatial correlation remaining in the data after fitting the MNL algorithm to the data. The median Moran’s I was 0.29 and 0.31 for HC and MCI groups respectively.
(PDF)

S5 Fig Partial correlation plots of the data for HC (top left) and MCI (top right) groups. Partial correlation plots of the residuals of the MNL algorithm for HC (bottom left) and MCI (bottom right) groups.
(PDF)

S6 Fig MNL results for 35 ROI application on AIBL case study data. To further assess the performance of the MNL algorithm, we applied our method on the same clinical diagnosis groups (Healthy Control (HC), mild cognitive imapired (MCI) and Alzheimer’s disease (AD)) on 35 ROI of the left hemisphere as done in Cespedes et. al. (2017) [35] on data from Australian Imaging, Biomarkers and Lifestyle (AIBL) study of ageing (https://aibl.csiro.au/). We found 83%, 84% and 85% of the connections in our network correctly match to those from the formal connectivity model, for the HC (left), MCI (middle) and AD (right) networks respectively. M. I. Cespedes, J. McGree, D. C. C., K. Mengersen, J. D. Doecke, and J. Fripp. A Bayesian hierarchical approach to jointly model structural biomarkers and covariance networks. In QUT ePrints: 112807, November 2017.
(PDF)
