# Supplementary material for: An efficient algorithm for estimating brain covariance networks
Source: PLoS One. 2018 Jul 12;13(7):e0198583. doi: 10.1371/journal.pone.0198583 (PMC6042721; doi:10.1371/journal.pone.0198583)
Supplement: S1 Table — A further simulation study was performed in order to assess the performance of the MNL algorithm (with γ set to 0.9) applied to simulated data with various levels of spatial dependence. Simulated data from the model (1) was generated with γ values {0.60, 0.70, 0.80, 0.90, 0.99}, σs2=1 and W = S1 as shown in Fig 1 of the manuscript. The sample size was set to N = 500. (PDF) [file pone.0198583.s001.pdf]

λ	Sensitivity	Specificity
0.60	0.97	0.66
0.70	0.96	0.67
0.80	0.98	0.67
0.90	0.97	0.67
0.99	0.53	0.53
