# Supplementary material for: An efficient algorithm for estimating brain covariance networks
Source: PLoS One. 2018 Jul 12;13(7):e0198583. doi: 10.1371/journal.pone.0198583 (PMC6042721; doi:10.1371/journal.pone.0198583)
Supplement: S2 Table — HC and MCI total number of network links derived by gLASSO for the range of 0.1 ≤ λ ≤ 1. This range encompasses a full networks where each ROI is connected with each other at 2,278 links (λ = 0.1), to the null networks with no connections (λ = 1). (PDF) [file pone.0198583.s002.pdf]

λ	HC (no.links)	MCI (no.links)
0.1	2278	2278
0.15	2278	2278
0.2	2278	2278
0.25	2278	2278
0.3	2278	2278
0.35	2278	2278
0.4	2146	2278
0.45	2018	2146
0.5	1778	2018
0.55	1777	2017
0.6	868	1911
0.65	629	1269
0.7	143	416
0.75	30	48
0.8	1	10
0.85	0	0
0.9	0	0
0.95	0	0
1	0	0
