# Supplementary material for: An efficient algorithm for estimating brain covariance networks
Source: PLoS One. 2018 Jul 12;13(7):e0198583. doi: 10.1371/journal.pone.0198583 (PMC6042721; doi:10.1371/journal.pone.0198583)
Supplement: S1 File — (PDF) [file pone.0198583.s003.pdf]

Here we provide a proof-by-cases that the parametrisation of the covariance matrix, $\sigma_s^2 Q$, is a positive definite matrix for all values of $0 \leq \gamma < 1$. If our covariance estimator is positive definite, then it satisfies a softer condition that covariance matrices must be positive semi-definite in order to be a valid covariance matrix [7].

There is a large body of literature which states that the maximum likelihood estimator of a parameter is a consistent estimator, and proofs of this fundamental result will be discussed at the end of this section.

SI Case 1: W has no neighbours

Theorem 8.4.6 in [3] (page 491) state that positive diagonal matrices are positive definite. We will rely on this theorem to show that when matrix W is a zero matrix, signifying no connections or spatial associations among regions, then $\sigma_s^2 Q$ is a positive definite covariance matrix.

When W is a zero matrix, Q^{-1} becomes a diagonal matrix with entries $\sigma_s^2/(1-\gamma)$, which is positive. Hence the matrix is positive definite [3].

SI Case 2: W has one neighbour

We first introduce a well known theorem on diagonally dominant matrices, as described in Cheney and Kincaid 2009 [6] (page 654) and elsewhere [4].

Theorem 1. *Matrix A is said to be strictly diagonally dominant if*

$$|a_{ii}| > \sum_{j \neq i} |a_{ij}| \qquad \forall i.$$

It follows that a strictly diagonally dominant symmetric real matrix with positive diagonal entries are positive definite [3, 4]. An alternative proof of this result can also be derived using Gerschgorin's theorem ([9] page 118-119). A well known property of positive definite matrices is that their inverse is also positive matrix [3] hence Q is also a positive definite matrix and $\mathbf{z}^T Q \mathbf{z} > 0$ holds for all real $\mathbf{z}$.

Without loss of generality, let W have a single neighbour and let that neighbour be $w_{34} = w_{43} = 1$, that is

$$W = \begin{bmatrix} 0 & 0 & 0 & 0 & \dots & 0 \\ 0 & 0 & 0 & 0 & \dots & 0 \\ 0 & 0 & 0 & 1 & \dots & 0 \\ 0 & 0 & 1 & 0 & \dots & 0 \\ \vdots & & & & & \\ 0 & \dots & & & & 0 \end{bmatrix}, \tag{1}$$

then matrix $\hat{W}$ is a zero matrix, whose only non-zero diagonal elements are $\hat{w}_{34} = \hat{w}_{43} = 1$.

Let Q_b^{-1} denote the bth vector of Q^{-1} and correspond to a row where W has no neighbours, that is a row of W with zero elements. Then

$$Q_b^{-1} = [0, 0, ..., 0, 1-\gamma, 0, \dots, 0]$$

and $1-\gamma$ is the bth element or the diagonals of Q^{-1}. Note: that all the other elements are zero, and $1-\gamma$ is the only non-zero element. Hence for rows $b = 1, 2, 5, 6, ..., K$, the absolute values of the diagonal elements are

greater than the sum of the off-diagonal elements.

The third and fourth rows of Q^{-1} are as follows

$$Q_3^{-1} = [0, 0, \gamma \sum_{i \neq 3}^{K} w_{3i} + 1 - \gamma, -\gamma, 0, \ldots, 0]$$
$$Q_4^{-1} = [0, 0, -\gamma, \gamma \sum_{i \neq 4}^{K} w_{4i} + 1 - \gamma, 0, \ldots, 0]$$

where the diagonal elements for rows 3 and 4 are $\gamma \sum_{i \neq 3}^{K} w_{3i} + 1 - \gamma$ and $\gamma \sum_{i \neq 4}^{K} w_{4i} + 1 - \gamma$ respectively.

As shown by matrix (3), since there is only one neighbour then $\sum_{i \neq 3}^{K} w_{3i} = \sum_{i \neq 4}^{K} w_{4i} = 1$ so the above simplifies to

$$Q_3^{-1} = [0, 0, 1, -\gamma, 0, \ldots, 0]$$
$$Q_4^{-1} = [0, 0, -\gamma, 1, 0, \ldots, 0]$$

Since $0 \leq \gamma < 1$, then the sum of the absolute values of the off-diagonal elements is γ for rows 3 and 4 and this value is less than one.

The results above show that in the case W has a single neighbour, then Q^{-1} is a positive definite matrix. Furthermore, this implies that Q is also positive definite and we can easily show that if we scale Q by a positive value, like σ_s^2, then we preserve the relationship $\mathbf{z}^T(\sigma_s^2 Q)\mathbf{z} > 0$ as $\mathbf{z}^T(\sigma_s^2 Q)\mathbf{z} = \sigma_s^2(\mathbf{z}^T Q \mathbf{z}) > 0$.

SI Case 3: W has $1 < n < K - 1$ neighbours

Assume connectivity matrix W has $1 < n < K - 1$ neighbours, then the precision matrix is

$$Q^{-1} = \begin{bmatrix} \gamma \sum_{i \neq 1}^{K} w_{1i} + 1 - \gamma & -\gamma w_{12} & \ldots & -\gamma w_{1K} \\ -\gamma w_{21} & \gamma \sum_{i \neq 2}^{K} w_{2i} + 1 - \gamma & \ldots & -\gamma w_{2K} \\ \vdots & \ldots & \ldots & \vdots \\ -\gamma w_{K1} & \ldots & \ldots & \gamma \sum_{i \neq K}^{K} w_{Ki} + 1 - \gamma \end{bmatrix}, \tag{2}$$

where the off-diagonal elements w_{mj} are equal to one if regions m and j are neighbours and zero otherwise. We begin by considering generic row m and consider the diagonal term and the sum of the off-diagonals at the extreme values of the range $0 \leq \gamma < 1$.

Consider generic row m, then its corresponding row vector is

$$Q_m^{-1} = \left[-\gamma w_{m1}, -\gamma w_{m2}, \ldots, \gamma \sum_{i \neq m}^{K} w_{mi} + 1 - \gamma, \ldots, -\gamma w_{mK} \right],$$

where $Q_{mm}^{-1} = \gamma \sum_{i \neq m}^{K} w_{mi} + 1 - \gamma$ for n connected regions, then the sum $\sum_{i \neq m}^{K} w_{mi} = n$.

Question: Is the nth row strictly diagonally dominant? Does the inequality

$$|n\gamma + 1 - \gamma| > | - n\gamma| \tag{3}$$

hold for $0 \leq \gamma < 1$?

When $\gamma = 0$ the above simplifies to $1 > 0$, so the inequality (3) holds.

Let's observe this expression as $\gamma \to 1$. Since the LHS of (3) is always positive because $\gamma \neq 1$, then (3) simplifies to $n\gamma + 1 - \gamma > n\gamma$. By combining the like-terms, the inequality becomes $1 - \gamma > 0$ and this holds for the range of γ. We conclude that when W has $1 < n < K - 1$ neighbours, Q^{-1} is positive definite. This implies that $\sigma_s^2 Q$ is also positive definite.

SI Case 4: W has $K - 1$ neighbours

This final case applies to matrix Q^{-1} where each region has $K - 1$ neighbours. Here matrix W looks like

$$W = \begin{bmatrix} 0 & 1 & 1 & 1 & \dots & 1 \\ 1 & 0 & 1 & 1 & \dots & 1 \\ 1 & 1 & 0 & 1 & \dots & 1 \\ & & & \ddots & & 1 \\ 1 & 1 & 1 & 1 & \dots & 0 \end{bmatrix},$$

and every diagonal of element of $\hat{W}$ will be $K-1$ and off-diagonals are zero. The expression for Q^{-1} becomes

$$\gamma(\hat{W} - W) + (1 - \gamma)\mathbb{I} = \begin{bmatrix} \gamma \sum_{i \neq 1}^{K} w_{1i} + 1 - \gamma & -\gamma & \dots & -\gamma \\ -\gamma & \gamma \sum_{i \neq 2}^{K} w_{2i} + 1 - \gamma & \dots & -\gamma \\ \vdots & & & \vdots \\ -\gamma & & \dots & \gamma \sum_{i \neq K}^{K} w_{Ki} + 1 - \gamma \end{bmatrix}$$

As each row has the same number of connections, then $\sum_{i \neq j}^{K} w_{ji} = K - 1$ for all j. The above simplifies to

$$\gamma(\hat{W} - W) + (1 - \gamma)\mathbb{I} = \begin{bmatrix} \gamma(K-2)+1 & -\gamma & -\gamma & -\gamma & \dots & -\gamma \\ -\gamma & \gamma(K-2)+1 & -\gamma & -\gamma & \dots & -\gamma \\ -\gamma & -\gamma & \gamma(K-2)+1 & -\gamma & \dots & -\gamma \\ \vdots & & & & & \vdots \\ -\gamma & -\gamma & & & \dots & \gamma(K-2)+1 \end{bmatrix}$$

For this matrix to be strictly diagonally dominant, and hence a positive definite matrix, then for each row we need to satisfy

$$|\gamma(K-2) + 1| > | - \gamma(K-1)|.$$

As it is straightforward to show that for $K = 2$ the corresponding precision matrix Q^{-1} is positive definite, this result has been omitted. For $K > 2$ the LHS is positive, hence we can simplify the above to $\gamma(K-2) + 1 > \gamma(K-1)$. As $0 \leq \gamma < 1$, then

$$\begin{aligned} \gamma K - 2\gamma + 1 &> \gamma K - \gamma \\ -2\gamma + 1 &> -\gamma \\ -\gamma + 1 &> 0 \end{aligned} \tag{4}$$

and this is true for all values of γ, as γ is bounded to be positive and less than one. Since we have shown that the covariance matrix $\sigma_s^2 Q$ is a positive definite covariance matrix for all possible number of neighbours, it remains to show that the estimation of positive definite covariance matrices by the maximum likelihood estimator (MLE) is consistent.

SI Literature which describe consistent properties of MLEs

There is ample literature which describe desirable properties of the multivariate normal density, such as the density being at least twice differentiable (continuity), well defined in the real domain and has a single global maximum (compactness) [10, 5]. These and other features have been described in literature, which go on to show that MLE for the normal density are consistent estimators [5, 1, 2, 8].

References

[1] T. Anderson. Asymptotically efficient estimation of covariance matrices with linear structure. *The Annals of Statistics*, pages 135–141, 1973.

[2] T. Anderson. An Introduction to Multivariate Analysis, 1984.

[3] H. Anton and R. C. Busby. *Contemporary Linear Algebra*. Wiley, 2003.

[4] K. Briggs. Diagonally Dominant Matrix, 1999.

[5] G. Casella and R. L. Berger. *Statistical Inference*, volume 2. Duxbury Pacific Grove, CA, 2002.

[6] W. Cheney and D. Kincaid. Linear algebra: Theory and applications. 2009.

[7] Y. Fujikoshi, V. V. Ulyanov, and R. Shimizu. *Multivariate statistics: High-dimensional and large-sample approximations*, volume 760. John Wiley & Sons, 2011.

[8] J. Rice. *Mathematical statistics and data analysis*. Nelson Education, 2006.

[9] Y. Saad. *Iterative methods for sparse linear systems*, volume 82. SIAM, 2 edition, 2003.

[10] D. Wackerly, W. Mendenhall, and R. Scheaffer. *Mathematical statistics with applications*. Nelson Education, 2007.
