# Supplementary figures and images for: An efficient algorithm for estimating brain covariance networks
Source: PLoS One. 2018 Jul 12;13(7):e0198583. doi: 10.1371/journal.pone.0198583 (PMC6042721; doi:10.1371/journal.pone.0198583)

Sparsity search in simulation study  $S_1$

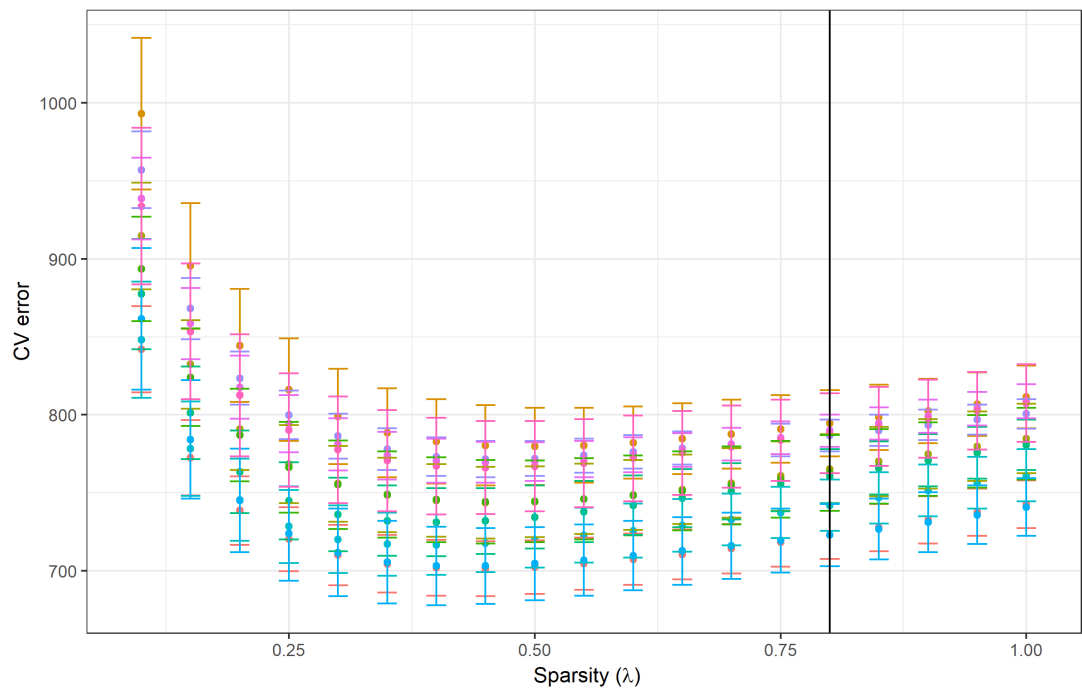

Sparsity search in simulation study  $S_2$

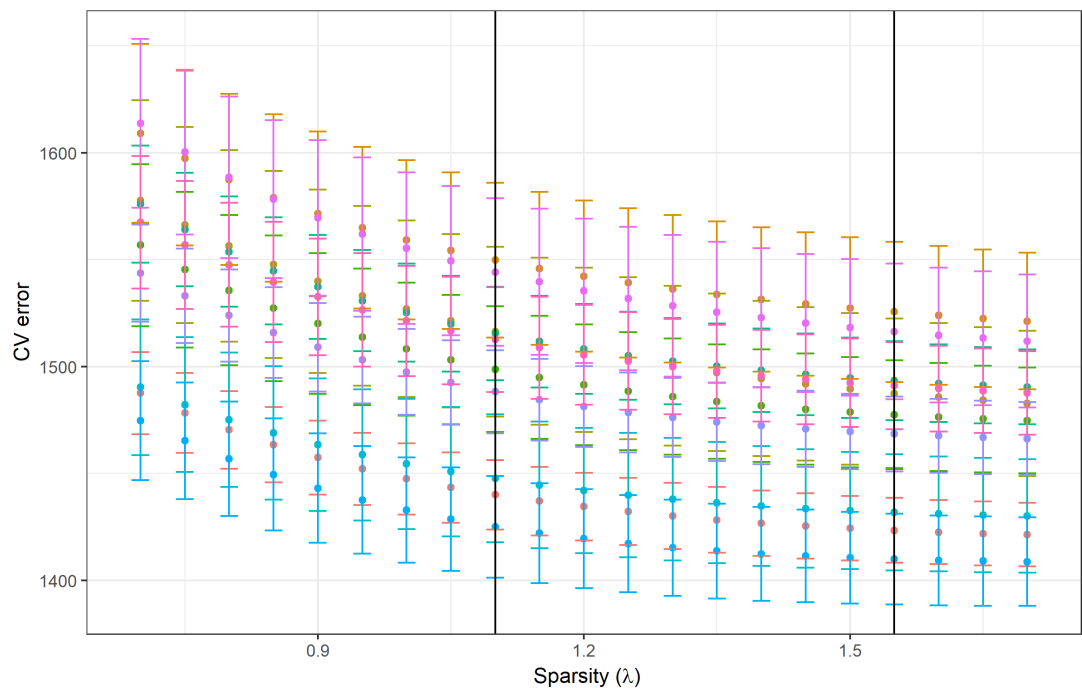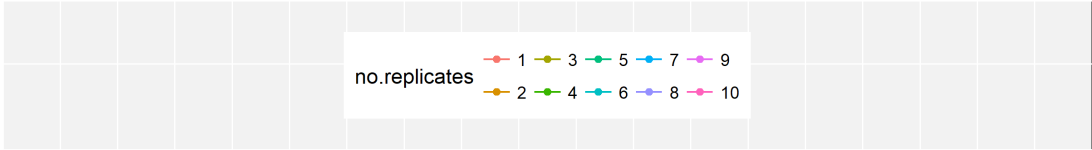

Supplement: S1 Fig — Cross validation mean and error intervals on simulated study for data simulated from binary matrices S1 (top) and S2 (bottom) for all ten replicates. Vertical black lines denote the optimal specificity and sensitivity λ trade-off as per manuscript Figs 2 and 3. Optimal value of λ is chosen such that the CV error is at its lowest. (PDF) [file pone.0198583.s004.pdf]

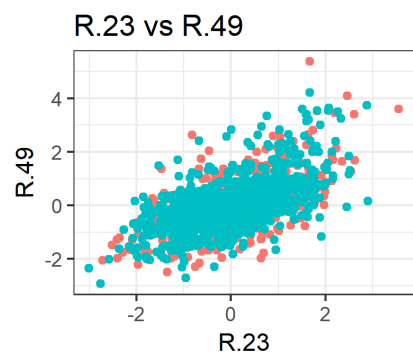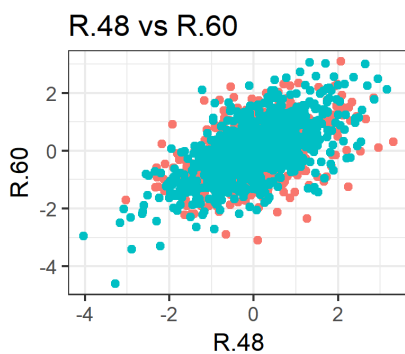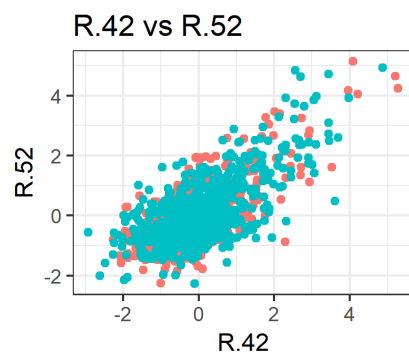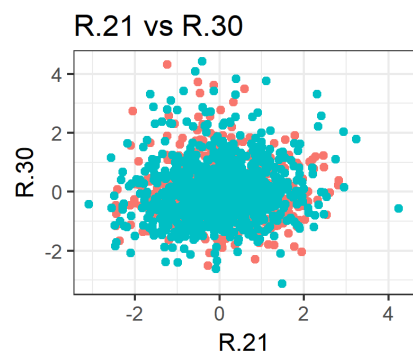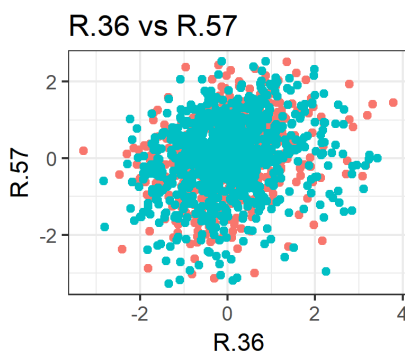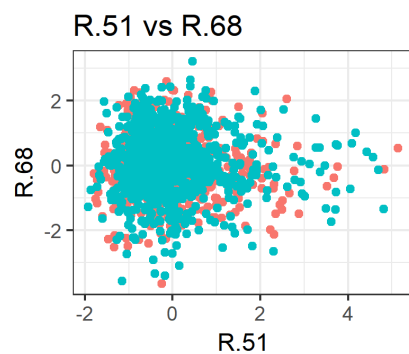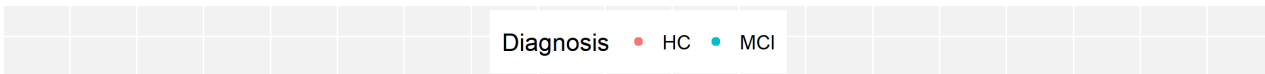

Supplement: S3 Fig — Pairwise scatter plots for selected HC and MCI ROIs. Top: ROI pairs denote linear association and correspond to link in estimated networks in Fig 4 of manuscript. Bottom: selected ROI pairs denoting an absent linear association and correspond to absent connections in Fig 4 of the manuscript. (PDF) [file pone.0198583.s006.pdf]

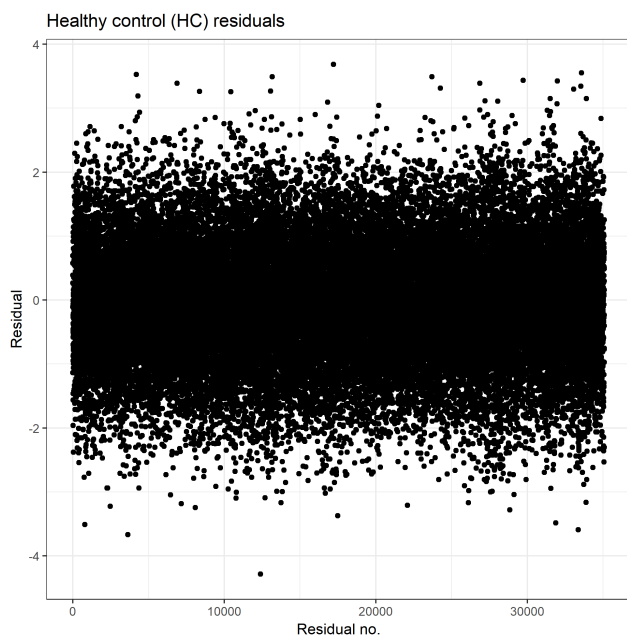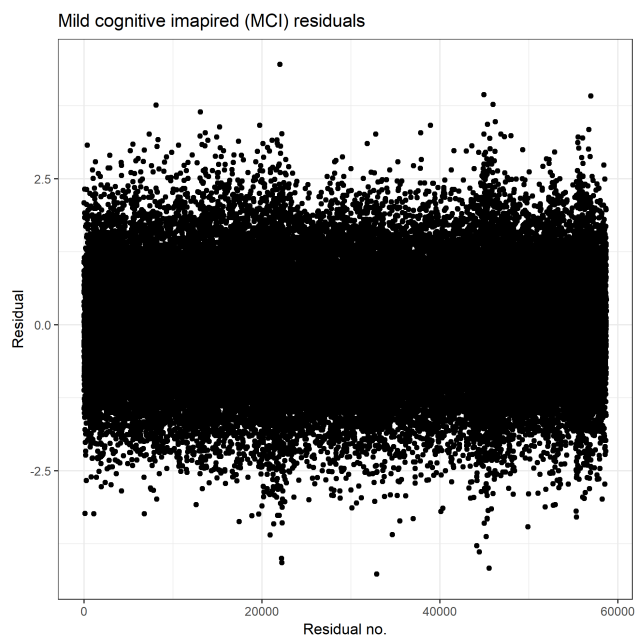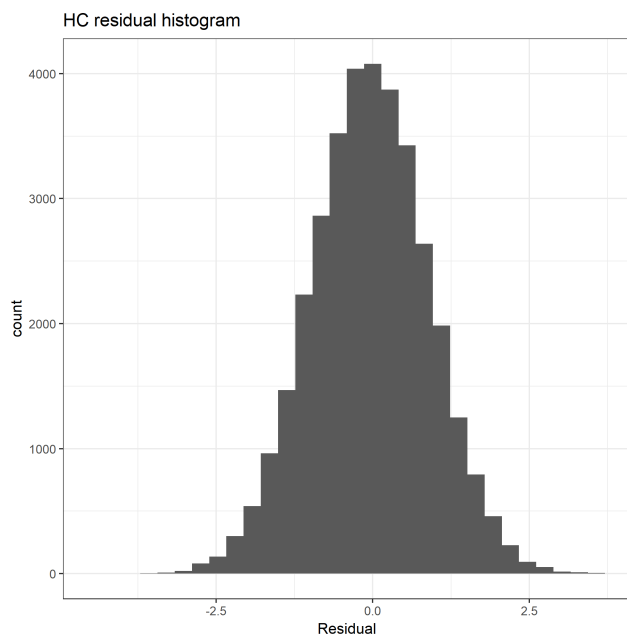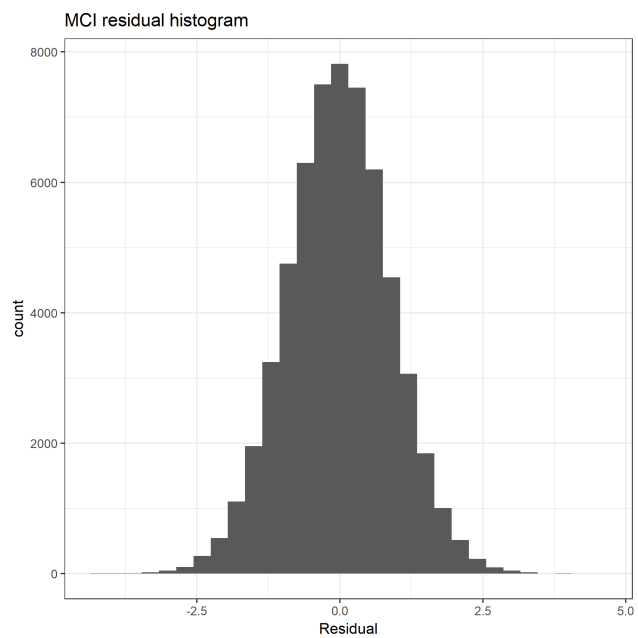

Supplement: S4 Fig — Top: residual scatter plots for HC (left) and MCI (right). Bottom: Residual histograms for HC (left) and MCI (right). As these residuals are approximately normal and the scatter plot shows no apparent deviations from zero, we conclude that there were no violations on our MNL model assumptions. Furthermore, we applied Moran’s I to each set of residuals on both HC and MCI groups to assess if there was still spatial correlation remaining in the data after fitting the MNL algorithm to the data. The median Moran’s I was 0.29 and 0.31 for HC and MCI groups respectively. (PDF) [file pone.0198583.s007.pdf]

HC transformed data

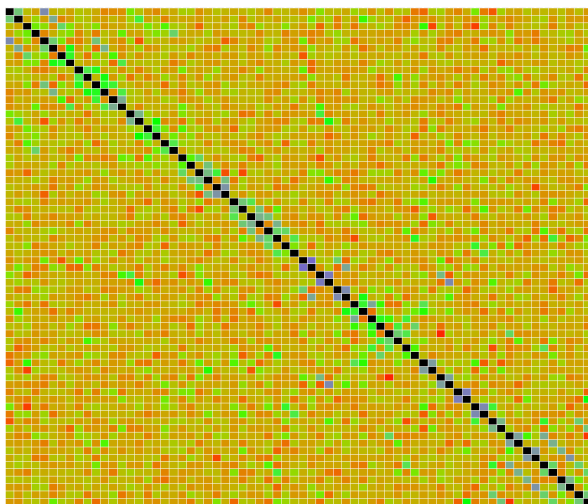

HC MNL model residual

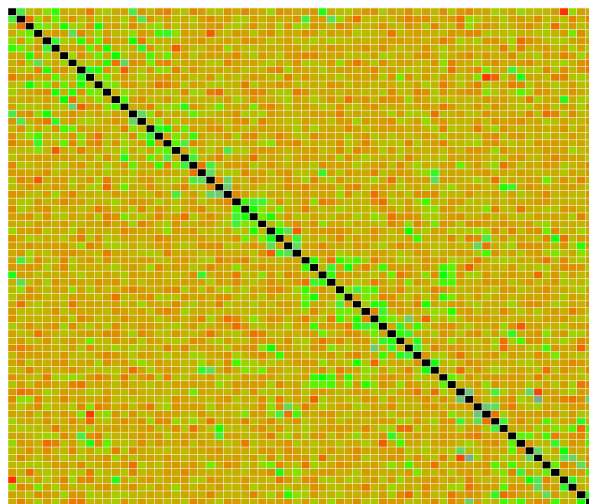

MCI transformed data

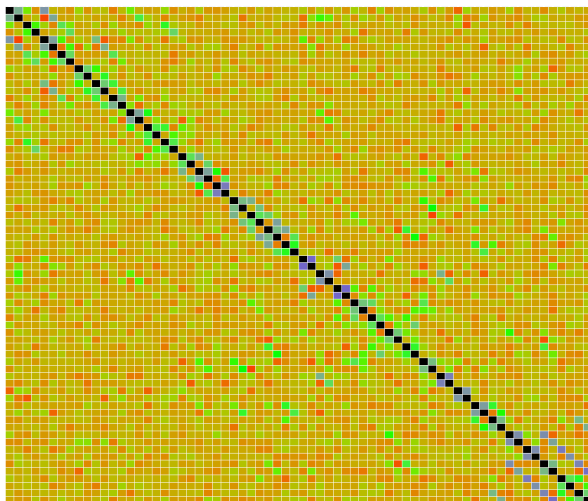

MCI MNL model residual

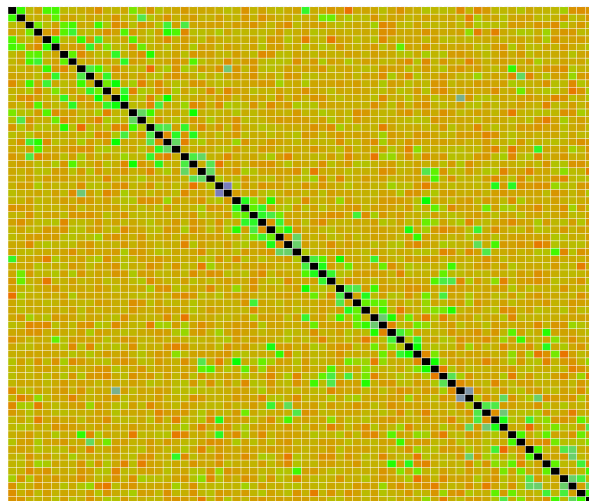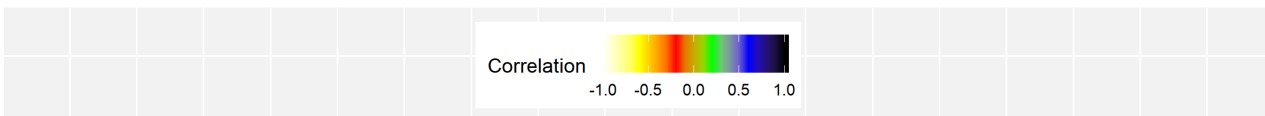

Supplement: S5 Fig — Partial correlation plots of the residuals of the MNL algorithm for HC (bottom left) and MCI (bottom right) groups. (PDF) [file pone.0198583.s008.pdf]

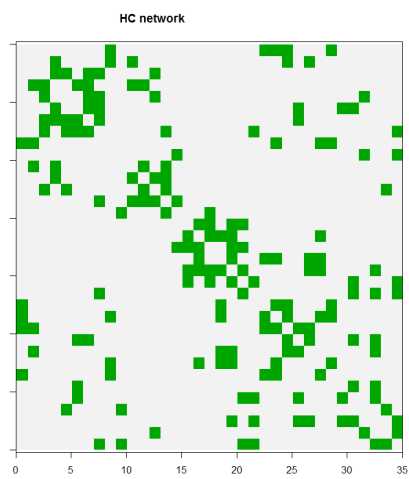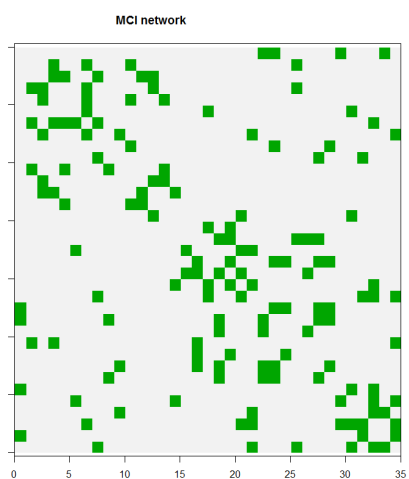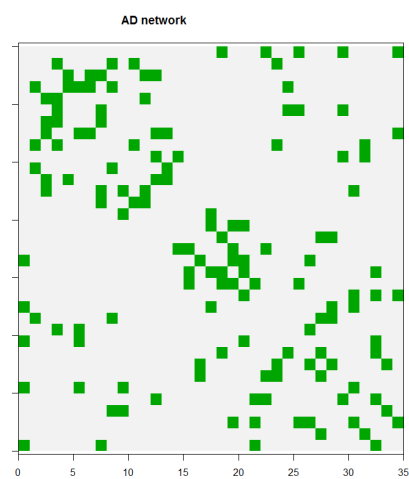

Supplement: S6 Fig — To further assess the performance of the MNL algorithm, we applied our method on the same clinical diagnosis groups (Healthy Control (HC), mild cognitive imapired (MCI) and Alzheimer’s disease (AD)) on 35 ROI of the left hemisphere as done in Cespedes et. al. (2017) [35] on data from Australian Imaging, Biomarkers and Lifestyle (AIBL) study of ageing (https://aibl.csiro.au/). We found 83%, 84% and 85% of the connections in our network correctly match to those from the formal connectivity model, for the HC (left), MCI (middle) and AD (right) networks respectively. M. I. Cespedes, J. McGree, D. C. C., K. Mengersen, J. D. Doecke, and J. Fripp. A Bayesian hierarchical approach to jointly model structural biomarkers and covariance networks. In QUT ePrints: 112807, November 2017. (PDF) [file pone.0198583.s009.pdf]
