# Supplementary material for: An efficient algorithm for estimating brain covariance networks
Source: PLoS One. 2018 Jul 12;13(7):e0198583. doi: 10.1371/journal.pone.0198583 (PMC6042721; doi:10.1371/journal.pone.0198583)
Supplement: S2 Fig — Sample covariance matrices for various sample sizes generated by S1 semi-sparse and S2 sparse matrix. (PDF) [file pone.0198583.s005.pdf]

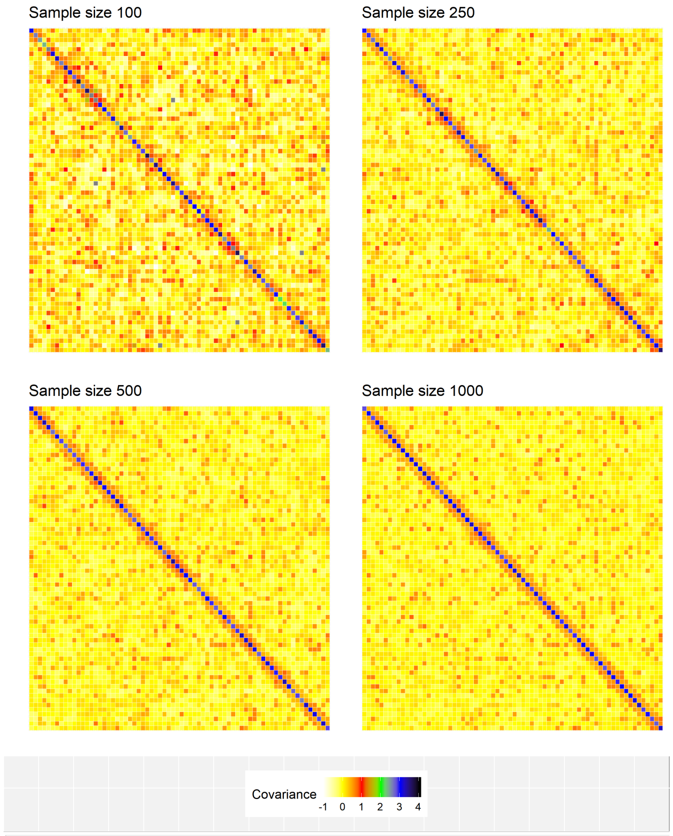
Sample size 100
Sample size 250
Sample size 500
Sample size 1000
Covariance
-1 0 1 2 3 4

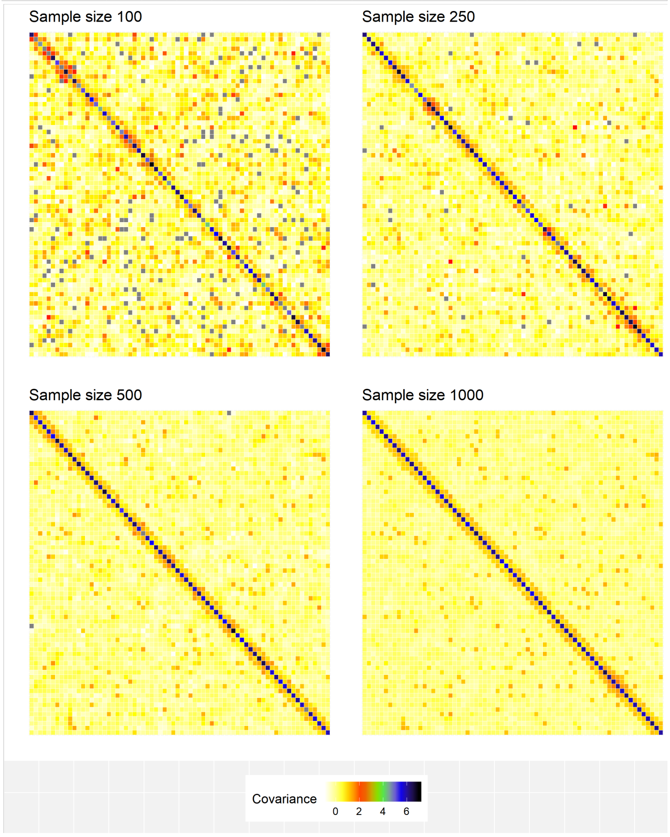
Sample size 100
Sample size 250
Sample size 500
Sample size 1000
Covariance
0 2 4 6
